# The effects of coenzyme Q10 on oxidative stress and heat shock proteins in rats subjected to acute and chronic exercise

**DOI:** 10.20463/jenb.2018.0019

**Published:** 2018-09-30

**Authors:** Ragip Pala, Fahrettin Beyaz, Mehmet Tuzcu, Besir Er, Nurhan Sahin, Vedat Cinar, Kazim Sahin

**Affiliations:** 1 Department of Movement and Training Science, Firat University, Elazig Turkey; 2 Department of Biology, Firat University, Elazig Turkey; 3 Department of Animal Nutrition, Firat University, Elazig Turkey

**Keywords:** exercise, coenzyme Q10, oxidative stress, heat shock proteins

## Abstract

**[Purpose]:**

The aim of the study was to determine the effects of dietary CoQ10 on serum biochemical parameters, lipid peroxidation, and HSP expression in the liver and slow-twitch muscles (soleus and gastronemius deep portion) of exercise-trained rats.

**[Methods]:**

A total of 42 Wistar albino rats were divided into six groups: 1) Control, 2) Coenzyme Q10 (CoQ10), 3) Chronic Exercise (CE), 4) CE + CoQ10, 5) Acute Exercise (AE), and 6) AE + CoQ10. The rats were subjected to the running test 5 days a week for 6 weeks after which CoQ10 was administered via the diet. AE (running on the treadmill until the rats were exhausted) was done on the last day

**[Results]:**

The results showed no significant difference in serum glucose and liver functions in any of the groups. However, CoQ10 and exercise treatment were found to lower cholesterol and triglyceride levels. Serum and muscle malondialdehyde (MDA) levels were found to be lower in the CE and CE + CoQ10 groups compared to the control group. The highest levels of HSP60, HSP70, and HSP90 in liver and muscle were found in the AE group, and the lowest levels were found in CE + CoQ10 group. CoQ10 supplementation reduced HSP expression in both CE-and AE-trained rats (P < 0.05).

**[Conclusion]:**

The results showed that CoQ10 supplementation could reduce MDA levels, protect against oxidative damage, and regulate HSP expression in CE-and AE-trained rats. CE and CoQ10 were shown to reduce oxidative stress synergistically.

## INTRODUCTION

The impairment of the antioxidant-mediated defense system during acute exercise (AE) training, which has been shown in muscles and the liver, results in the increase of reactive oxygen species (ROS) and inflammation markers^1^. At the end of the maximal exercise, this condition of the muscle tissue causes free radicals to multiply, leading to lipid peroxidation of the membranes, and an increase in the abundance of macrophages and white blood cells^2^. Exercise can increase the use of oxygen by over 200 times and increases the relaxation levels of working muscle fibers^3^. During exercise-training, muscle mitochondria mediates an increase in superoxide production with the increase in oxygen flow^3,4^. Increased amounts of free radicals associated with excess oxygen consumption is counteracted by a defense system containing enzymatic and non-enzymatic antioxidants. Excessive exercise is manifested as muscle fatigue and muscle damage known as oxidative stress due to imbalance between ROS and antioxidants^5^. In a previous study, it was shown that regular exercise provides many benefits, while excessive exercise increases oxidative damage by increasing the ROS formation^6^. Studies have been conducted on both marathon runners and experimental animals in order to eliminate or mitigate such negative effects caused by exercise. These studies were mostly based on the removal of oxidative stress and were conducted with reinforcing substances which are thought to have strong antioxidant properties.

Heat shock proteins (HSPs) are molecular chaperones that help fold proteins into their original conformation, restore function, and contribute to the reduction of cellular damage^7-9^. They have many functions in maintaining intracellular integrity via protection, repair, and even regulation of cell death signaling^10^. It is well documented that the expression of HSPs, particularly HSP70, has been found to be increased in the mammalian skeletal muscle under stress conditions caused by exercise^8,11,12^. It has been reported that HSP70 expression is upregulated immediately after thermal stress in the soleus muscle^13^. The upregulation of HSPs may be responsible for cytoprotection via a mechanism capable of reestablishing protein homeostasis against numerous stressors, including exercise^14^. Chronic exercise (CE) may improve the overall health; however, a single bout of inadequate exercise may lead to oxidative stress and muscle damage^15,16^. Taken together, these studies suggest that the expression of HSPs could be considered important for protection from and repair of skeletal muscle after exercise-induced stresses.

Coenzyme Q10 (CoQ10), naturally found in mitochondria, is a potent antioxidant that is endogenously synthesized and is soluble in fat^17^. Due to its antioxidant properties, it is able to effectively inhibit the oxidation of fat, protein, and DNA in the body^18^. CoQ10 suppresses lipid peroxidation, and thus, oxidative stress by suppressing the activity of enzymes involved in ROS production^19,20^. One of the most important functions of CoQ10 is to serve as an electron carrier during oxidative phosphorylation by diffusing to the phospholipid layer of the cell membrane via a unique chain structure in mitochondria^21^. In addition, it plays an important role in energy production from carbohydrates and fatty acids in cells^22^. Besides, CoQ10 decreases serum cholesterol levels by suppressing cholesterol synthesis in the liver^23^.

Oxidative stress, which occurs during extreme exercise, lowers CoQ10 levels in mitochondria^24^. Thus, excessive free radicals are created that accumulate in the muscles, leading to fatigue and muscle damage in the body^25^. It has been reported that CoQ10 supplementation is highly effective in transporting and using oxygen required at the cellular level^26^ as well as in eliminating muscle damage and fatigue^27^. Therefore, the aim of the present study was to determine the effects of dietary CoQ10 on serum biochemical parameters, lipid peroxidation, and the expression of heat shock proteins (HSP60, HSP70, and HSP90) in the liver and muscles of acutely and chronically exercised rats.

## METHODS

### Animals

This study was carried out by following the appropriate ethical rules for animal welfare and animal rights, after approval (No: 2014/01-07) from the Firat University Ethics Committee for Animal Experiments (Elazig, Turkey). A total of 42 male Wistar rats (8 weeks old, 180 ± 20 g weighted, 7 in each group) were housed in a controlled room with a 12/12 hour light/dark cycle at 22 ± 2 ° C and 55 ± 5% relative humidity in specially prepared and daily cleaned cages, and supplied with rat chow and water ad libitum.

The rats were divided randomly into the following 6 groups: (1) Control (C); rats received basal diet and were not exercised, (ii) CoQ10; rats received basal diet supplemented with CoQ10 and were not exercised, (iii) CE; rats received basal diet and were exercised (30 m/min, gradient 0 %, 30 min; exercise was performed for 5 days per week for 6 weeks), (iv) CE + CoQ10; rats received basal diet supplemented with CoQ10 and were subjected to CE (v) AE; rats received basal diet and were exercised (30 m/min, gradient 0 %, 30 min for 5 days per week for 6 weeks and exhaustion exercise was also performed); and (vi) AE+ CoQ10; rats received diet supplemented with CoQ10 and were subjected to AE. CoQ10 was administered daily for six weeks as an oral supplement by a gastric tube at a dosage of 300 mg/kg body weight. The selection of the dose (300 mg/kg b. wt.) was based on previous studies where this dosage elicited a significant antioxidant effect in rats^28^. After exercise training of rats in the AE groups, exhaustion exercise (making the rats run rats until they are exhausted and collecting serum and tissue samples immediately) was performed.

### Exercise protocol

The rats were subjected to a motor-driven rodent treadmill at a 0 % gradient (MAY-TME, Commat Limited, Ankara, Turkey). The treadmill was supplied with an electric shock grid on the rear barrier to provide exercise motivation to the rats. All exercise tests were done during the same time period of the day to diminish diurnal effects (11.30–13.30 hours). The rats in the CE groups were familiarized by treadmill exercise over a 5-d period such as: 1st day 10 m/min, 10 min; 2nd day 20 m/min, 10 min; 3rd day 25 m/min, 10 min; 4th day 25 m/min, 20 min; and 5th day 30 m/min, 0 % grade, 30 min at high intensity^29^. Thereafter, animals were exercised at this level for 6 weeks, 5 days/week. Exhaustion was defined as the inability of a rat to right itself when being laid on its side. All animals were fasted for 12 h before death.

### Sample collection

At the completion of the exercise program, rats were decapitated by cervical dislocation, and blood, slow-twitch muscles (soleus and gastronemius deep portion), and liver samples were taken. Blood samples were centrifuged at 5000 rpm at 4 °C for 10 minutes in a refrigerated centrifuge (Universal 320R, Hettich, Germany) with gliotic biochemical tubes (Standard plus & Medical Co., Ltd., Germany) to isolate serum. All the samples were stored at -80 °C (Hettich, Germany) for further analyses.

### Laboratory analysis

Levels of serum glucose, cholesterol, triglyceride, aspartate aminotransferase (AST), and alanine aminotransferase (ALT) were analyzed with an autoanalyzer (Samsung LABGEO PT10, Samsung Electronics Co, Suwon, Korea). Repeatability and device/method precision of LABGEO PT10 was established according to the IVRPT06 guideline. The malondialdehyde (MDA) levels of serum, liver, and muscle were measured by high-performance liquid chromatography (HPLC; Shimadzu, Tokyo, Japan) using a Shimadzu UV–vis SPD-10 AVP detector and C18 ODS-3.5 μm, 4.6 mm × 250 mm column^30^.

### Analysis of gene expression

For total RNA extraction, 50 mg of tissue sample was homogenized using a Tissue Ruptor (Qiagen, Venlo, Netherland) homogenizer attached with a probe in 600 μL of 1:100 beta-mercaptoethanol in RLT buffer. The samples were then centrifuged for 3 min. The supernatant was collected and mixed with one volume of 70 % ethanol. The total RNA was isolated from 50 mg of liver and muscle samples using the RNeasy total RNA isolation Mini Kit (Qiagen, Hilden, Germany) according to the manufacturer's instructions. After isolation, amount and the quality of total RNA were determined using nanodrop spectrophotometry (Maestrogen, USA). Occurrence of any RNA degradation was checked with agarose gel electrophoresis. cDNA synthesis was carried out by using 500 ng of total RNA and oligo(dT)18 primer using a commercial First Strand cDNA Synthesis Kit (MBI Fermentas, USA) as per the manufacturer's instructions. Gene expression of desired proteins were determined with real time PCR by mixing 1 μl cDNA and 5 μl 2x SYBR Green Master-mix (Qiagen Fast Start Universal, SYBR Green Master Mix).

### Real time PCR analysis

Predeveloped TaqMan primers and probes set for HSP60, HSP70, and HSP90 were designed at ABI based on gene sequence information obtained from GenBank (Qiagen Lot no: 20111214039, 20120813061, and 20110719024, respectively). The glyceraldehyde 3-phosphate dehydrogenase gene (GAPDH), which has been widely used as a reference gene in rats^31^, was used as internal control (Qiagen Lot no: 20130917040). Then primer pairs (Table 1) were added at final concentrations of 0.5 mM to a final volume of 10 μl. The real time PCR program of the quantitative PCR (Light Cycler 480 II, Qiagen, Tokyo, Japan) was arranged as follows: initial denaturation at 95 °C for 10 minutes, denaturation at 95 °C for 15 s, annealing at 65 °C for 30 s, and extension at 72 °C for 15 s with 40 repeated thermal cycles measuring the green fluorescence at the end of each extension step. PCR reactions were carried out in triplicates and specificity of PCR products was verified by melt analysis. Also, negative controls lacking template were used in all reactions. The relative expression of genes with respect to GAPDH was calculated with efficiency corrected advance relative quantification tool provided by the software (http://www.qiagen.com/geneglobe Data Analysis Center). Gene expression profiles were indicated as ΔCT values. Gene expression fold changes were presented relative to the control and calculated using the 2 -ΔΔCT method. The differential gene expression was rated in pairs with a fold change cut off of 2 and significance value of *P* < 0.05^31^.

**Table 1.** Effects of CoQ10 on biochemical parameters in rats subjected to AE and CE

Variables	Control	CoQ10	CE	CE + CoQ10	AE	AE + CoQ10
Glucose (mg/dL)	105.7 ± 5.68	107.2 ± 5.01	104.8 ± 5.91	107.3 ± 4.96	106.0 ± 3.30	105.3 ± 6.32
Cholesterol (mg/dL)	80.6 ± 3.12^a^	71.1 ± 2.91^b^	69.7 ± 0.85^b^	68.4 ± 1.64^c^	69.3 ± 0.62^b^	68.7 ± 4.30^b^
Triglyceride (mg/dL)	93.8 ± 2.85^a^	83.8 ± 1.78^b^	84.7 ± 2.58^b^	72.0 ± 2.16^d^	84.3 ± 2.42^b^	74.3 ± 2.23^c^
AST (U/L)	230.8 ± 19.44	221.5 ± 17.82	225.3 ± 26.34	221.9 ± 11.2	219.3 ± 15.42	222.0 ± 17.78
ALT (U/L)	92.0 ± 8.89	91.0 ± 6.26	85.7 ± 6.15	90.4 ± 7.89	92.3 ± 6.05	97.0 ± 7.02

Data is given as mean ± standard error. Different superscripts (a–d) indicate group mean differences (P < 0.05). Control: Rats fed a basal diet; CoQ10: rats fed a basal diet supplemented with CoQ10; CE: rats fed a basal diet and subjected to CE; CE + CoQ10: rats fed a basal diet supplemented with CoQ10 and subjected to CE; AE: rats fed a basal diet and subjected to AE; AE + CoQ10: rats fed a basal diet supplemented with CoQ10 and subjected to AE.

### Statistical analysis

Data was assessed using ANOVA in SPSS software program (Version 22.0; Chicago, IL, USA). Comparisons between groups were analyzed by the Tukey post hoc test. Data was expressed as group mean and standard error of the mean (SEM). *P* < 0.05 was considered to be significant.

## RESULTS

### Serum biochemical parameters

Serum glucose, cholesterol, triglyceride, AST, and ALT concentrations are shown in Table 1. Serum cholesterol and triglyceride levels in exercised or CoQ10 supplemented rats were significantly different from the control group rats (*P* < 0.0001, for both); however, differences in the serum glucose, AST, and ALT levels were not significant (*P* > 0.05). The serum cholesterol and triglyceride concentrations were decreased in the CE + CoQ10 group (*P* < 0.0001, for both; Table 1).

### Serum and tissue MDA levels

As shown in Table 2, in general, serum, liver, and muscle MDA levels were found to be decreased by CE and CoQ10 supplementation in rats (*P* < 0.05). However, an increase in MDA level was detected with AE (*P* < 0.001). The lowest serum, liver, and muscle MDA levels were found in the CE + CoQ10 group, while the highest serum, liver, and muscle MDA levels were found in the AE group (*P* < 0.001, respectively; Table 2).

**Table 2.** Effects of CoQ10 on oxidative stress in rats subjected to acute and chronic exercise training rats

Variables	Control	CoQ10	CE	CE + CoQ10	AE	AE + CoQ10
Serum MDA (nmol/mg protein)	0.88±0.04^c^	0.61±0.03^d^	0.55±0.02^d^	0.43±0.03^e^	1.37±0.05^a^	0.91±0.03^b^
Liver MDA (nmol/mg protein)	101.4±2.24^c^	95.1±2.46^d^	96.1±2.04^d^	87.2±1.94^e^	117.7±2.55^a^	108.3±2.8^b^
Muscle MDA (nmol/mg protein)	85.2±3.11^c^	61.3±2.05^d^	59.6±1.82^d^	53.0±1.50^e^	97.7±2.22^a^	93.6±1.43^b^

Data is given as mean ± standard error. Different superscripts (a–d) indicate group mean differences (P < 0.05). Control: Rats fed a basal diet; CoQ10: Rats fed a basal diet supplemented with CoQ10; CE: Rats fed a basal diet and subjected to chronic exercise; CE + CoQ10; rats fed a basal diet supplemented with CoQ10 and subjected to chronic exercise; AE: Rats fed a basal diet and subjected to acute exercise; AE + CoQ10: Rats fed a basal diet supplemented with CoQ10 and subjected to acute exercise.

### Expression of heat shock proteins

**Fig. 1.**
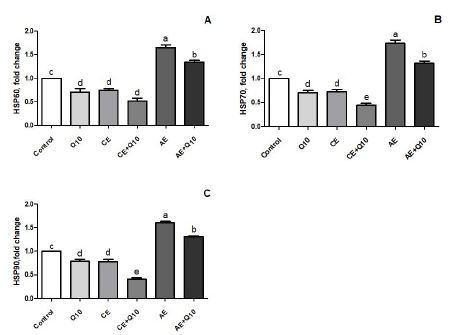
Effects of CoQ10 on the expression of liver heat shock proteins (HSPs) in rats subjected to CE and AE training. Control: rats fed a basal diet; CoQ10: rats fed a basal diet supplemented with CoQ10; CE: rats fed a basal diet and subjected to CE; CE + CoQ10: rats fed a basal diet supplemented with CoQ10 and subjected to CE; AE: rats fed a basal diet and subjected to AE; AE + CoQ10: rats fed a basal diet supplemented with CoQ10 and subjected to AE. Gene expression was analyzed by RT-PCR. Different superscripts (a–d) indicate group mean differences (P < 0.05).

**Fig. 2.**
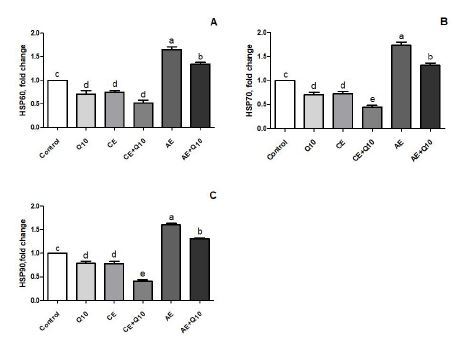
Effects of CoQ10 on expression of muscle heat shock proteins (HSPs) in rats subjected to CE and AE training. Control: rats fed a basal diet; CoQ10: rats fed a basal diet supplemented with CoQ10; CE: rats fed a basal diet and subjected to CE; CE + CoQ10: rats fed a basal diet supplemented with CoQ10 and subjected to CE; AE: rats fed a basal diet and subjected to AE; AE + CoQ10: rats fed a basal diet supplemented with CoQ10 and subjected to AE. Gene expression was analyzed by RT-PCR. Different superscripts (a–d) indicate group mean differences (P < 0.05).

Expression of HSP60, HSP70, and HSP90 genes in liver and muscles of rats in AE group were higher than those in the other groups (Fig 1A, B, C and 2A, B, C; *P* < 0.001). CoQ10 administration significantly decreased the expression of HSP60, HSP70, and HSP90 genes in liver and muscle (*P* < 0.05). The decrease in the expression of HSP60, HSP70, and HSP90 in the liver and muscle of the rats in the CE + CoQ10 group was more prominent than in the other groups (*P* < 0.05; Fig 1 and 2).

## DISCUSSION

In the present study, the effects of CoQ10 supplementation on serum biochemical parameters, liver functions, lipid peroxidation, and expression of HSP60, HSP70, and HSP90 in the liver and muscle tissues of acutely and chronically exercised rats have been revealed. CoQ10 and AE/CE training were found to lower cholesterol and triglyceride levels (Table 1). These results show that during exercise training CoQ10 does not instantly mediate carbohydrate or lipid metabolism in the organism. CoQ10 is known as a redox molecule with intracellular antioxidant efficacy that is biochemically present in biological tissues both in reduced and oxidized form interacting with oxygen-derived radicals and singlet oxygen, thereby preventing the initiation of lipid peroxidation and damaging biomolecules^32,33^. Also, CoQ10 is an ongoing antioxidant which plays a vital role in the regeneration of other antioxidants when exposed to plasma oxidants, although it is present in low concentrations compared to other antioxidants (e.g. α-tocopherol)^34,35^. Furthermore, CoQ10 has been reported to have roles in membrane fixation, cell signaling, gene expression, cell growth, and supervision of apoptosis^36^. Similar to our research, Pala et al. found that CoQ10 supplementation in exercised rats led to a decrease in cholesterol and triglyceride levels^37^. Kim et al.^38^ have previously reported that there was a significant difference in serum cholesterol and triglyceride levels, and Yoon and Park^39^ have reported that there was a significant difference in serum triglyceride levels in exercised rats supplemented with antioxidant agents such as soy isoflavones, L-carnitine, and vitamin E. However, Díaz-Castro et al. found that there was no significant difference in cholesterol levels, but triglyceride levels were decreased in CoQ10 supplemented amateur male runners^40^. It has been also been shown that during exercise, CoQ10 tend to improve skeletal muscle activity and triglyceride capacity that could be responsible for its ergogenic effects^41^.

Increased lipid peroxidation and tissue damage in overt exercises are associated with increased oxidative stress^42,43^. MDA, the product of lipid peroxidation, is a dialdehyde that is the result of peroxidation of unsaturated fatty acids and is indicative of oxidative damage^44,45^. In the present study, the control group exhibited decreased levels of serum, liver, and muscle MDA after CoQ10 supplementation and CE (Table 2). Díaz-Castro et al. stated that CoQ10 supplementation in amateur male runners reduced oxidative stress and inhibited muscle damage^40^. In another study, Pala et al. reported that CoQ10, which acts as an antioxidant, suppresses oxidative stress in rats who have been practicing running exercises and consuming CoQ10^37^. Gul et al. also reported that CoQ10 supplementation inhibited oxidative stress in 15 healthy and sedentary males who were subjected to Wingate tests^46^. Kon et al. examined the effects of CoQ10 supplementation in rats subjected to exercise and found that CoQ10 is useful in reducing physical exercise-induced muscle damage^28^. Ostman et al. have reported that CoQ10 may be effective on the alleviation of oxidative stress in moderately trained healthy men^47^. In addition, Pala determined that end-of-match MDA levels of elite boxers were significantly decreased^48^, and Pala et al. found that MDA levels caused due to oxidative stress decreased post-match in the coaches of Turkish boxing team^49^.

In the present study, AE induced significant increases in the expression of HSP60, HSP70, and HSP90 in the liver and muscles. However, CE induced a decrease in the HSP60, HSP70, and HSP90 expression in the liver and muscles of rats (Fig. 1 and Fig. 2). We also demonstrated that CoQ10 supplementation decreased HSP60, HSP70, and HSP90 expression in the liver and muscle of acutely and chronically exercised rats. It was seen that the greatest reduction in expression of HSPs was in the chronic exercised and CoQ10-supplemented group. To the best of our knowledge, no previous studies related to investigative the effects of CoQ10 supplementation on the expression of HSPs in rats acutely and chronically exercised rats have been conducted. However, there have been studies on the effects of exercise on expression of HSPs^50-53^. HSPs are known to act as cell defense mechanism, and they play an important role in the synthesis and repair of proteins^50^. HSPs are expressed especially in oxidative stress conditions (e.g. exhaustive physical exercise)^51^, and mitochondria are effectively protected by members of the HSP family including HSP60, HSP70, and HSP90^52,53^. Previously, the expression of the stress proteins were found to increase in rats subjected to running training^52,53^. Moreover, exercise-induced HSP responses are dependent on exercise intensity and duration, reflecting a physiological response to heat shock and oxidative stress^54^. Similar to our findings, it was reported that AE increased HSP60 mRNA, HSP90 mRNA and protein levels, and AE-induced oxidative stress (indicated by increased levels of HO-1 mRNA and protein), and HNE protein adducts^55^. In addition, Starnes et al.^56^ indicated that after three months of exercise for 5 days/week, HSP70 expression was not increased in the liver of aged rats, although it was significantly elevated in young rats^56^. In a previous study, it was shown that administration of CoQ10 decreased the levels of colon tissue MDA, HSP70, tumor necrosis factor-α (TNF-α), and interleukin-1β (IL-1β) in an ulcerative colitis model of rats^57^. There are also studies on the effects of antioxidants like Q10 on HSPs. For example, Oksala et al. reported that antioxidant supplementation effectively suppressed the heat shock protein expression in the liver of exercised rats^58^. Wu et al. reported that lipoic acid significantly decreased the levels of HSP70 expression in different muscle types in heat-shock induced rats^59^.

A decrease in lipid peroxidation and heat shock proteins in rats in the present study could have been due to positive effects of CoQ10 and/or CE, alleviating the negative effects of AE. In the present study, CE and dietary CoQ10 inclusions resulted in a synergic effect. More specifically, the combination of CoQ10 and CE provided the lowest levels of lipid peroxidation and HSP expression. It is apparent that a combination of dietary CoQ10 and CE offers a feasible way to reduce the increase in lipid peroxidation and HSP expression^29,37^. CoQ plays a role in inhibiting lipid peroxidation by either scavenging ROS directly or in conjunction with α-tocopherol^60^. Shimomura et al.^61^ have reported that intravenous CoQ10 supplementation suppressed the upregulated muscle damage markers (creatine kinase: CK, and lactate dehydrogenase: LDH) in rats following downhill running. The CoQ10 deficiency in skeletal muscle led to a spectrum of clinical manifestations and was it is suggested that this also leads to a secondary impairment of mitochondrial fatty acid oxidation^62^. In a previous study, it has been reported that CoQ10 supplementation increases CoQ concentration in muscle cell membranes and reduces AE-induced muscular injury by enhancing cell membrane stabilization^63^.

## CONCLUSION

In conclusion, these results showed that CoQ10 supplementation did not affect serum glucose levels and liver function, but reduced cholesterol and triglyceride concentrations in acutely and chronically exercised rats. In addition, CoQ10 showed protective effects against oxidative stress caused by AE by lowering MDA levels in acutely exercised rats. However, while AE increases oxidative stress, CE decreases oxidative stress by reducing lipid peroxidation. This effect has also been demonstrated by the regulation of the expression of heat-shock proteins in the liver and muscle tissues of CoQ10-fed rats. Meanwhile, CE and CoQ10 have been shown to reduce oxidative stress synergistically.
